# The Good, the Bad and the Blend: The Strategic Role of the “Middle Leadership” in Work-Family/Life Dynamics during Remote Working

**DOI:** 10.3390/bs11080112

**Published:** 2021-08-17

**Authors:** Paola Spagnoli, Amelia Manuti, Carmela Buono, Chiara Ghislieri

**Affiliations:** 1 Department of Psychology, University of Campania Luigi Vanvitelli, 81100 Caserta, Italy; carmela.buono@unicampania.it; 2 Department of Education, Psychology and Communication, University of Bari Aldo Moro, 70121 Bari, Italy; amelia.manuti@uniba.it; 3 Department of Psychology, University of Turin, 10124 Torino, Italy; chiara.ghislieri@unito.it

**Keywords:** remote work, work-life interface, middle leadership, technostress, workaholism, gender differences

## Abstract

The ongoing epidemiological crisis has suddenly steered us towards a new futuristic work scenario in which most service sector employees work remotely, which could be a permanent reality for most service sector employees. This paper focuses on the strategic role that leadership could play in the radical change process that is taking place in work environments. Particular attention was paid to the role of ‘middle managers’ who perform an important function as a link between the strategic vision of top management and the workforce. In addition, special attention was paid to gender differences in work-life dynamics, which are particularly relevant in countries with traditional cultural identities. As this is a conceptual contribution, the most recent studies on this specific role of middle managers have been taken into account and embedded in the current scenario. Therefore, the main contribution in terms of originality was that the current review aimed to leverage such a legacy of knowledge and create a system of evidence-based practical implications for effectively supporting change in organizational culture through the identification of the most appropriate middle management leadership models for remote working that could prevent and/or limit any psychosocial risks (e.g., workaholism and technostress) and longer-term outcomes such as sustainable work-life interface.

## 1. Introduction

Apart from the obvious health consequences, the epidemiological emergency following the SARS-COV-2 virus outbreak had a significant social and economic impact, with the expansion of ‘forced’ remote working for a large proportion of workers being one of the main reasons. According to recent data published by the Politecnico’s Telework Observatory in Milan [1] before the pandemic, there were 570,000 teleworkers in Italy. However, a report published in 2020 showed that during the lockdown 94% of public administrations, 97% of large companies and 58% of small and medium enterprises switched to telework, and the number of teleworkers increased to 6.58 million [2]. It is expected that remote working will evolve and completely transform working practices, affecting 51% of large companies.

In this scenario, researchers’ attention immediately turned to understanding the benefits and risks that this “new normal” would bring, including the implications for the future of the labor market. However, some preliminary conclusions from the literature are still contradictory. On the one hand, recent research has shown that telecommuting could offer better work-life balance opportunities, higher productivity and greater organizational flexibility. On the other hand, it may lead to stressful experiences, social isolation, work-life conflicts and difficult time management [3].

Nevertheless, this experience of “forced” transition could be an opportunity to conduct a “mega-experiment” in this field and draw useful conclusions for the development of new interventions to improve the quality of personal and professional life. Among the many salient aspects, the following could be considered in particular: rethinking the spatial and temporal boundaries between the spheres of personal life and work life; the need to review the communication and strategic management of people through leadership focused on sustainability; and finally, improving the relationship between workers and the progress of new technologies. The question of the difficulty of maintaining the boundaries between work and family life could recall phenomena already highlighted in the literature, such as overwork culture, workload and work addiction [4]. This pressing intrusion of work into people’s lives could create a new work-life interface that can only be partially interpreted in light of some of the consolidated models of work-life balance (and/or conflict) available in the literature. Moreover, during the epidemiological crisis, the difference between those who already had psychosocial resources useful for a positive work-life balance before the crisis and those who did not have these resources and experienced the negative effects of remote work seems to intensify [5]. In this context, gender differences play a particularly important role [6].

In light of these findings, scholarly contributions have suggested that leader’s behaviors can play an important role in mitigating the risks of negative consequences of remote work and promoting the sustainability of work processes [7,8]. Previous studies emphasized the role of leadership in supporting work-life balance [9] and, in particular, the role of compassionate leadership that is able to recognize employees’ concerns, thereby reducing stress and generating positive emotions [10]. However, traditional leadership models also need to be adapted to the new work contexts and new forms of leadership (e.g., “e-leadership”) need to be considered and “tested” in practice [11].

Based on these considerations, this paper focused on the strategic role of leadership in the ongoing change processes. In particular, the focus was on “middle managers” who perform an important liaison and mediation function between the strategic vision of top management and the line. Precisely because of the organizational position these professional figures occupy in the corporate chart, metaphorically represented in the literature as filling a sandwich [12], middle managers experience the weight of change management to a greater extent than others. Even though there are a large number of scholarly articles on leadership, no studies have been conducted on the further commitment that middle managers need to make in the “New Normal” scenario.

The organizational impact of the digitization of work, amplified by the radical change triggered by the pandemic, allows a comparison with the challenges experienced by middle managers in virtual organizations. However, there are some clear differences between the latter and what middle managers experience in the current scenario. On the one hand, middle managers in virtual organizations were able to rely on a “digitization culture” that provided them with psychological and material resources to cope with the difficulties of managing remote work. On the other hand, the abrupt change in work modalities as a result of the pandemic left middle managers unable to cope with the challenges they often faced (e.g., difficulties in maintaining work-life boundaries, difficulties in controlling processes and outcomes, difficulties in managing employees, etc.), leading to negative feelings of stress and pressure. Accordingly, the literature on middle managers’ leadership in virtual teams has confirmed that these contexts, due to their inherent characteristics (physical distance, communication difficulties, etc.), rely on a very clear and defined structure of roles, responsibilities and task assignments, which should be agreed upon and cannot be carried out impromptu.

In many cases, the pandemic has impacted work environments that were not prepared to “hold their fire.” In this sense, this paper aims to fill this research gap by exploring the difficulties and risks associated with the leadership role of middle managers in the post-pandemic organizational context (workaholism, technostress), as well as possible opportunities associated with the enhancement of the role of middle managers, with particular reference to the issue of work-life interface.

## 2. Work-Life Interface between Resources and Demands

Organizational studies have focused on work-life dynamics for decades because it is related to well-being and quality of life and work [13,14,15]. Even though much research is now available, the relationship between life domains is a complex and multifaceted issue. Indeed, it is subject to contextual conditions (welfare resources, labor market conditions, etc.), but also to the different value systems of generations. For these reasons, the topic requires continuous in-depth study and research that can capture different reading angles.

Scholars have highlighted the importance of the work-family interface (and more and more studies have extended to the work-life interface), including in terms of its close relationships with personal and organizational well-being and productivity [16,17].

Moreover, this is an issue that is particularly challenged by the Covid 19 containment measures. As highlighted in some position papers from an industrial and organizational psychology perspective [18,19], remote working has clearly posed problems of work-life balance, particularly for women [20], raising issues that will continue to arise, albeit with obvious differences, in a future where remote working is chosen by companies more frequently than in the past, albeit to a certainly less pervasive extent and under less dramatic contextual conditions.

Several authors have helped to advance scholarly knowledge about the work-life interface by proposing coherent theoretical models. Some of the most valuable models are the Theory of Resource Conservation (COR) [21] and Job Demands-Resources Theory (JDR) [22]. Another important reference is the perspective model of work-life interface resources [23], which is a synthesis of COR and JDR in the field of work-life interface.

The COR model [21] describes how individual motivation drives people to maintain their current resources and strive for new resources. This theoretical model is very useful for understanding the relationship between work and family stress. Indeed, the resource management that workers use in the work context may influence the availability of residual resources that can be used in the family context. In this sense, investing a greater number of resources in work may compromise the management of personal resources in the family, so that these resources may not be sufficient to meet the demands posed by the context.

In addition, JD-R theory is based on the idea that all professional realities are characterized by job resources and demands, and that the management of these elements can have positive and negative effects on well-being, through the activation of two simultaneous processes called the energy process and the motivational process, respectively [22]. Job demands refer to the organizational, psychosocial aspects of work that require cognitive and emotional effort and are associated with certain psychological and social costs. By job resources, on the other hand, we mean the physical, psychosocial and organizational aspects of work that are conditions for achieving work goals, stimulate growth, learning and personal development, and reduce work demands and associated psychophysiological costs [22].

The integrated model proposed by Ten Brummellhuis and Bakker [23], based mainly on the COR—and the JDR model, states that work-family conflict, thought of as a form of inter-role conflict with incompatible pressures from the work and family domains [24,25], is related to a situation in which contextual work demands reduce outcomes at home through a loss of personal resources. When participation in one role improves the quality of performance in the other role [26], the enrichment occurs in the same direction (work-family), when work resources increase outcomes at home through a process that increases personal resources. In the home-work direction, the demands at home may result in a loss of resources that is reflected at work (conflict), while the resources at home result in an increase in personal resources, which results in enrichment.

Despite the considerable evidence gathered through these perspectives in practice, we believe that these theoretical models need to be re-read in light of the unprecedented changes brought about by the epidemiological crisis scenario, in order to plan and develop HRM intervention practices that can best be adapted to current working conditions and workers’ needs.

In this framework, leadership could be both a requirement and a resource, as it can produce positive and negative outcomes in terms of organizational behavior (stress and/or well-being). Similarly, when focused on work-family issues, leadership can be a source of work-family conflict and/or enrichment. In this perspective, leadership is considered as a key factor in improving quality of life in the workplace, and this is especially true for leaders themselves, regardless of their position (top or middle) in the organizational chart. Drawing attention to middle managers, as this was the specific objective of the paper, we addressed the role that this position plays in the current challenging scenario of change, arguing that due to their function within the organizational chart, there is a need to further analyze the challenges and opportunities that could arise from a strategic management of this position.

## 3. The Challenges of the “Sandwich” Middle-Management’s Leadership in Managing Work-Life Interface

Common sense generally associates the term “leadership” with those professionals who occupy a top management position. However, organizations are very complex systems with varying degrees of responsibility and control, so leadership resides at different levels of the hierarchy.

One of the most important levels of the organizational chart is middle management, which is the leadership responsible for coordinating between the top level of management and the major functions of the lower levels. This role may require exceptional leadership skills, as middle managers are called upon to manage two-way exchanges between managers and employees, receiving guidance from top management while actively using their leadership skills to guide their colleagues at lower levels of the organizational chart. In both cases, however, the leadership effectiveness of middle managers is challenged by many contextual conditions, both at the sociocultural level (e.g., digitization of work, changes in workforce composition, etc.) and at the organizational level (e.g., organization of work processes, work-life balance programs, division of labor, etc.), which consequently can affect important individual and organizational performance (e.g., work addiction, off-the-job behavior, productivity, work-related stress, etc.).

Despite the radical changes affecting workers during the epidemiological crisis, a return to “normality” in the post-crisis period would hopefully be an opportunity to learn from experience, capitalize on the positive legacy of this situation and redesign work processes to improve individual and organizational quality of life.

This goal certainly requires a strategic rethinking of the boundaries between the times and spaces of paid work and the rest of life, the prevention of modern and extreme forms of heavy work investment (e.g., workaholism), the correct use of new technologies and the building of an organizational culture in which relationships are positive and focused on the common goal. In this context, the function of leadership, particularly the leadership “received” and exercised by middle managers, as a link between organizational strategies and the extensive network of relationships between the various work groups and their members, is of fundamental importance.

Given the central role played by middle managers, this topic has received increasing attention in recent decades, both in the academic literature and in professional practice. A separate strand of research has been opened, the so-called “middle management perspective” [27].

In the management context, the term “middle management” is used to refer to the group of managers located in the hierarchy below the top managers and above the first level of management [28]. The special role that middle managers occupy in organizations is not exclusively related to the position they occupy in the organizational chart, but rather to their privileged access to top management and, therefore, to their accurate knowledge of operations. This combination makes them intermediaries between the organization’s strategy and its daily activities [29]. From a purely functional point of view, this group of managers can include different types of mid-level professionals, such as general line managers (heads of departments or strategic business units), functional line managers (vice presidents of specific functions) and team or project-based managers (team leaders of strategic project managers). This different categorization, and thus heterogeneity in the skills and responsibilities of middle managers, depends on the different size of the organization (small or medium, large or multinational), the type of organization (e.g., profit or nonprofit) and the organizational culture that inspires processes and practices (e.g., family business, lean organization, hierarchical organization).

However, a common feature of this professional function is that middle managers are responsible for managing people, as they are responsible for the work of their employees. Since they are actively involved in the processes of strategy making [30,31], they are subject to constant change and therefore must themselves act as agents of change to their employees [32]. As a result, middle managers are often pressured on the one hand by the leadership of senior management, which sets visions and strategies, and on the other hand by employees, who demand innovative and sustainable leadership skills from them to help them cope with the challenges and demands of the work context. This is further complicated by the increasing use of remote working devices and modalities, which can undoubtedly have a positive impact on speeding up processes and improving performance, even if they pose a potential challenge to workers’ wellbeing. For example, previous research has shown a relationship between remote work and technostress [33,34], work-life balance [35] and burnout [36], but also a positive relationship with performance [37].

In light of the above, the only element that could actually make the difference in terms of employee acceptance/resistance to change is a deeply rooted organizational culture, i.e., the awareness of sharing common meanings and patterns of behavior that reveal a vision for the future and encourage coping with the difficulties that change inevitably brings [38].

Based on these assumptions and considering the position and delicate role played “between” organizational charts, middle managers were considered strategic agents of change, called to “understand, unify and transmit organizational culture” [39] (p. 393). Accordingly, they could have access to and manage very valuable intangible resources that could help shape and orient employees’ organizational behavior. Following some of the authoritative contributions in this field [40,41], middle managers could have the prerogative of managing attention, meaning, trust and the self. Due to their credibility and formal role, they could play a strategic role in spreading a new organizational culture. However, they could help create a compelling vision of the present and future, communicate the importance of the vision to employees and provide positive feedback. However, they could also act as a positive role model by demonstrating the need for behavioral change and making decisions that could have a collective impact on a larger number of people, thereby reinforcing employees’ perceptions of the interdependence of the individual and the organization [42].

## 4. Exploring the Light and the Dark Side of the Leadership

Middle managers, similar to all key positions within the organization, must contend with a critical, if intangible, resource that is further challenged by the current scenario: Leadership.

The concept of leadership has changed a lot over the years and various concepts of leadership have been proposed. Until the 1970s, most accepted models of leadership were anchored in the concept of transactional leadership, in which leaders were viewed as professionals who maintained the quality of interaction with their employees through their behavior and used the levers at their disposal to promote motivation. The main features of transactional leadership are the use of reward systems to maintain employee motivation and the implementation of corrective actions (related to one’s leadership style) when the goal is not achieved.

Later, thanks to the seminal work of Burns [43], the concept of transformational leadership prevailed, which emphasizes the leader’s symbolic behavior, visionary and inspirational messages, nonverbal communication, appeal to values, stimulation and motivation at both intellectual and emotional levels. In the words of Burns [43], the transformational leader is someone who recognizes the needs of followers and knows how to transform his followers into new leaders.

In the last 20 years, the topic of authentic leadership, a concept that dates back to the 1990s of the 20th century, has gradually gained prominence in organizational research and practice [44]. Interest in authentic leadership has grown in response to anxieties associated with social change, major failures and the never-ending financial crisis with its correlates of uncertainty. In this context, authenticity is a concept that offers a hopeful alternative to the widespread fear and discontent in the world of work and responds to the need for truly responsible individuals in key positions, i.e., leaders who are able to combine their work with qualities such as integrity, transparency, courage and optimism [45].

The introduction of models focusing on the ethical dimension is attributed to the incompleteness of transformational leadership in terms of moral issues, transparency and values [44,46,47]. Authentic leadership is only effective when it is supported and integrated into the organizational context in which it is expressed, which must be characterized by access to information, availability of resources, perceived support and equal opportunities for learning and development. Therefore, a virtuous cycle must be set in motion in which authentic leadership fosters an inclusive organizational culture, which in turn fosters the possibility of authenticity in the relationship between leaders and followers and the possibility of attracting and developing this type of leadership [48].

Due to the recent radical changes in the workplace caused by rapid technological advancements, a new concept of leadership has emerged, that of electronic leadership (e-leadership). E-leadership was explored in the late 1990s with the rapid emergence of advanced information technologies (AIT) such as the Internet, email, video conferencing, WhatsApp, virtual teams and virtual learning platforms. Electronic or e-leadership is not only an extension of traditional leadership, but also represents a critical shift in the way leaders and followers relate to each other within organizations and with stakeholders [49], making it imperative for leaders to change their practices [50]. Avolio defined e-leadership “as a process of social influence mediated through AIT to bring about a change in attitude, feelings, thinking, behavior and/or performance in individuals, groups and/or organizations” [51] (p. 617).

Even though many areas of work were not yet ready, the COVID-19 pandemic and its associated social constraints suddenly drove change through increased teleworking, resulting in a sharp break in previously established leadership practices. As Contreras and colleagues [52] noted, e-leadership implies the development of certain skills to improve organizational functioning in virtual work environments [53]. For e-leaders, the known social skills, such as the characteristics of effective face-to-face communication, may not be sufficient to lead in virtual environments, where these characteristics need to be complemented by the skills of managing different virtual communication platforms. Liu and colleagues [54] noted that e-leadership can lead to alienation and chaos if this process is not properly addressed by leaders and is only used to issue instructions. In virtual or remote work environments, leaders should display a more inclusive leadership style [55]. For e-leaders, social skills, such as the characteristics of effective face-to-face communication, may not be sufficient to lead in virtual environments [53]. The e-social environment is the second important characteristic of e-leadership [53], i.e., creating a positive work atmosphere with a sense of connectedness with the group to enhance communication and collaboration through digital communication methods. The e-social characteristics of e-leadership can successfully prevent the isolation of team members [56].

Even though several studies examined the role of leadership in employees’ work-life balance, highlighting the importance of the enrichment between life and work that could result from perceived support and, therefore, the reduction of conflicts, research in this area, in line with [57], requires a change of pace, considering not only positive leadership but also the dark side of leadership, i.e., toxic, destructive and abusive behaviors [58,59]. A growing number of studies point to the existence of these forms of leadership, which are fostered by certain organizational practices and, in particular, encouraged by the “always-on” culture. Therefore, there is no shortage of studies that have investigated the impact of leadership on expected outcomes, both at the individual and organizational level, considering leadership (transformational, authentic, etc.) primarily as a resource [60].

In his recent review of studies conducted between 1980 and 2015, Arnold [61] highlighted that transformational leadership appears to positively predict measures of well-being and negatively predict measures of unhappiness, however, noting that recent studies suggest that this relationship is not always direct and that it is important to delve into the effects of mediation and moderation. Positive leadership can establish transparent communication with followers aimed at understanding conciliation problems and requests for extra work [62,63]. The role of leadership is also critical to organizational outcomes and employee well-being in times of crisis [64], such as the Covid 19 epidemic, where leadership effectiveness in managing remote work was critical [19].

However, in organizations, there is not only “good leadership”. In this regard, there is growing scientific evidence that managers and supervisors can perform their roles in dysfunctional ways that have significant negative consequences for individuals and organizations [65,66,67,68,69]. As highlighted earlier, positive and negative aspects of expressing leadership in organizational life are intertwined, even when expressed by the same person. Therefore, it is crucial to understand the interplay between the light and dark sides of the leadership experience.

Destructive leadership negatively affects people’s well-being and can lead to physical health problems, emotional damage and psychological stress [70]. Consequences include feeling constantly belittled and reminded of past mistakes or having one’s opinions devalued [71], as well as work tension and emotional exhaustion [72], lower job satisfaction and commitment [71,73], decreased family well-being [74], workaholism [7], absenteeism, turnover intentions and low performance [75], and physical health problems [75,76,77,78].

Following the integrated theoretical model proposed by Ten Brumhelluis and Bakker [23], destructive leadership can be counted among work demands. Accordingly, “bad leadership” creates tension, enforces an environment of control, leads to intensification of work to avoid negative feedback [73], to the possible escalation of micromanagement behaviors, resulting in people being trapped in a tiring, unsatisfying and detrimental work life. Controlling and micromanaging behaviors tend to be associated with destructive leadership in which the authoritarian component and/or abusive trait predominates [75].

However, other forms of leadership that are not intentionally abusive can also be detrimental to well-being. Laissez-faire forms of leadership, for example, can move toward drifting, a true form of abandonment; the relationship loses its value, the bond provided by the meaning of the work is lost, people are disoriented and experiences of dissatisfaction and detachment ensue. Some studies have already highlighted the challenging role of destructive leadership in emergency remote work [58] by increasing the risk of technostress [33] or internet addiction [79], which is an increased risk in contexts characterized by always-on cultures without boundaries (neither spatial nor temporal) between work and the rest of life [14,80].

Following this overview of the framework of leadership models and the new work scenario, it seems obvious that a very important prerequisite for the successful assumption of the middle leadership role is that middle managers should be able to manage their leadership within the organization. As highlighted earlier, middle managers can provide leadership for their employees but at the same time should be able to manage the leadership exercised by the top management. Furthermore, if this double burden is difficult in “normal” times, it could become an even greater burden in this “new normal” scenario, exacerbated by the demands of telecommuting.

Nevertheless, especially with regard to remote work, there are some challenges that middle managers must overcome. As active leaders of their employees, they need to communicate differently when interacting with some employees in person and with others virtually, they need to define and adopt new behaviors that can be observed by all in order to promote social cohesion and build trust in their teams [81]. The issue of communication and social interaction in remote work is fundamental. In fact, isolation in remote work can lead to lower engagement, as social interaction in the workplace provides socialization, participation and a sense of belonging and trust [82]. In addition, informal interactions that occur more spontaneously between employees who work in presence do not occur as easily in a virtual environment. Managers therefore need to find new approaches to create them, as people are likely to work remotely as well as in the present [81].

As “recipients” of the leadership exercised by top management, middle managers must cope with higher cognitive and concrete workloads resulting from the borderless management of work that digital devices make possible. Moreover, they might also experience emotional pressure due to excessive control by top management if the latter adopts a more directive and transactional leadership style [83]. In light of the above, existing leadership models are probably not sufficient to represent the transformation that this function is undergoing in order to be effective and make remote work profitable and sustainable through goal-oriented work management and a non-invasive, empathic and participative communication style.

## 5. Workaholism and Technostress’ Risks for Middle Managers during Remote Working

Even though the link between the leadership role, in this case with specific reference to the position and responsibilities of middle managers, and the work-life interface seems obvious, it could also be mediated by some workplace risks particularly associated with remote working during the pandemic, such as a dysfunctional form of high work engagement (e.g., workaholism) and technostress. From a legal perspective, among the “new” risks faced by workers in these conditions (compared to the “traditional” ones), there are some that are specifically psychosocial in nature. Workaholism and technostress can be considered as work-related risks arising from the culture of high work investment [4] and the massive use of ICT (information and communication technologies)—in the relationship between paid work and the rest of people’s lives [84].

The phenomenon of workaholism affects some people who devote their energies and leisure time exclusively to work life, in an uncontrollable and compulsive manner such as a true behavioral addiction [85], which means a significant impairment of psychophysical health [86]. Nevertheless, workaholism does not seem to be recognized by society today as a pathological malaise because it is related to work, which is indispensable and of general interest.

In this context, Robinson [87] refers to workaholism as “the well-dressed addiction” because it is underestimated by society as a legitimate behavior or activity that is socially accepted and even encouraged in some work contexts. In studies of the development of workaholism, it has traditionally been described as a personal disposition similar to other personality traits such as perfectionism or conscientiousness.

However, a consistent research approach has recently emphasized the role that some work factors may play in the development of workaholism. Work overload [88] and a climate of overwork [89] have been identified as possible triggers of the phenomenon. It is also likely that excessive and unbalanced use of new technologies to manage remote work may play a crucial role in triggering the spiral of mechanisms leading to workaholism. However, studies in this regard are still quite scarce, and there is a need to contribute to the expansion of knowledge about workaholism in the context of mass remote work. Some studies have highlighted that transformational leadership can protect against the occurrence of workaholism [8], while abusive and “laissez-faire” leadership can promote the occurrence of workaholism [90].

Even though they are supposed to make our lives and work easier through faster communication, faster computing and the ability to reach anyone anywhere [91], new technologies can lead to negative effects such as stress, discomfort and anxiety due to the use of the Internet, email, instant messaging and smartphones [92]. Symptoms associated with technostress may include anxiety, behavioral tension, feelings of exhaustion, mental fatigue, poor concentration, physical illness and insomnia, while the main consequences are reduced productivity, job (dis)satisfaction, lower organizational commitment and increased absenteeism and turnover [33]. A recent paper reported that authoritarian leadership style may increase the harmful effects of workaholism on the occurrence of technostress, which is likely due to the invasiveness and over-control of work tasks [59].

What would be interesting to investigate, and this would also be an innovative aspect as there are no contributions in this sense in the literature, is the influence that “top management” leadership may have on the role of the middle manager, a role that is already known in the literature to be a risk for workaholism [8], and how the leadership practices of the middle manager may be intrinsically harmful or protective against the risks of workaholism, technostress and the work-life interface.

A recent study has highlighted how technostress can be linked to the phenomenon of workaholism, although the relationship between workaholism and ICT has previously been mainly related to the phenomenon of techno-addiction (an uncontrollable “pressure of duty” combined with anxiety when ICT is not used, leading to excessive use of ICT for long periods of time) [92] or the fact that workaholism can lead to intensive smartphone use [93].

However, since workaholism implies a combination of worry and a desire to always stay connected to work, it is interesting to observe its relationship with technostress and, in particular, to examine what kind of relationship exists between workaholism and technostress. One of the hypotheses is that there is a reciprocal and bidirectional relationship between workaholism and technostress, i.e., that the anxiety resulting from the use of new technologies for work may favor the occurrence of workaholism and that the latter, in turn, generates a higher level of technostress.

Regarding workaholism, empirical evidence did not reveal significant gender differences, while regarding technostress, several studies reported that women may be particularly affected by it [94], which exacerbates the inequalities to their disadvantage. Therefore, the relationship between workaholism and technostress should be examined from the perspective of gender differences. In general, the perspective of gender differences is particularly important for the study of the work-life interface in remote work [58].

## 6. A Proposal for a Model, Looking at Gender Differences

In light of these considerations, the analysis of the relationship between middle management leadership and these new psychosocial risks (technostress and workaholism) becomes particularly interesting, especially with regard to HRM interventions that could improve workers’ performance and well-being. Accordingly, it could be argued that some kind of toxic/abusive leadership practices exerted by top management on middle managers could influence the occurrence of some negative organizational behaviors (e.g., workaholism and technostress) among this target group, which would affect the entire workforce. In fact, middle managers would in turn exert negative forms of leadership on their employees and in some cases reproduce pressure, stress and in the worst cases work addiction/workaholism.

**Figure 1:**
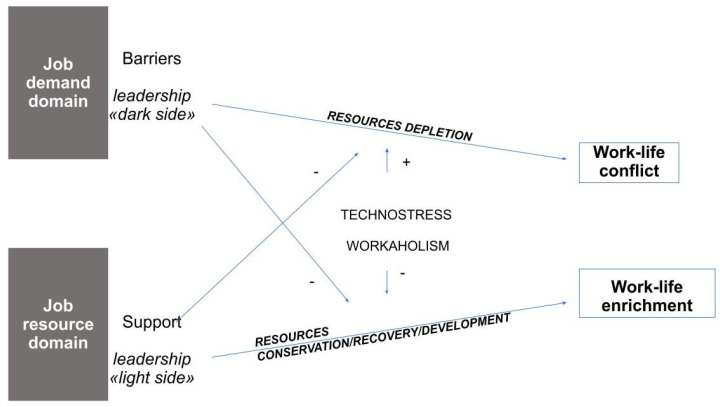
The conceptual model.

The dark side of leadership associated with these psychosocial risks can manifest itself in forms of excessive control: the spillover of work on employees’ personal lives restricts the “right to disconnect” [95], a fundamental element in activating the recreational experiences necessary for overall well-being. An illustration of the conceptual model we propose can be seen in Figure 1.

In this scenario, the issue of gender differences is central, as mentioned above, as it touches on these issues “since time immemorial” (e.g., the work-family interface), with a very large literature already existing and growing to reflect rapid demographic and labour market changes. The pandemic and, in particular, the mitigation measures have exacerbated (and are likely to continue to exacerbate) the gender gap, so there is a need to focus on gender differences.

In particular, the 2021 report on Gender Equality in European Union [20] highlighted a number of issues where the pandemic has placed women in greater difficulty: domestic violence and the difficulty of escaping it, exacerbated by domestic confinement and the feeling of not being able to access help (despite ad hoc measures); more severe psychological and/or health consequences (higher levels of anxiety, insomnia or sleep disturbances, post-traumatic stress symptoms, negative changes in cognition and emotion and also hyperarousal and exhaustion [58,96,97]; the prevalence of women in occupational fields that are associated with health risks or are in crisis, while employment fields that are not in crisis are typically “male-dominated”; and, partly due to the previous point, higher female unemployment and greater difficulty in finding a new job. The prevalence of women’s involvement in work-life balance challenges and ‘return to home’ risk (higher percentage of women teleworking yesterday, today and perhaps in the future, with likely career risks) deserves particular attention.

As noted earlier, the work-family interface is a classic issue of interest in the field of gender inequality [98]. Women’s participation in the labor market since the 1970s has challenged the traditional division of gender roles into breadwinner (male) and homemaker (female) [99,100,101]. However, the shift in roles towards a better balance is still ongoing, with large differences between countries (traditionalist vs. egalitarian gender culture) and generations [102,103]. In many countries, e.g., in Mediterranean European countries such as Italy, women are still considered as the main responsible for the household and family care, even if they are employed or have a career. Data confirm that women spend more hours than men on activities, to mention just a few examples, such as cleaning in the home, preparing meals, supervising children’s homework, caring for elderly relatives [104,105]. Managing work and family life simultaneously without traditional institutional support [106] is a major challenge for women [20] and increases the burden of care [107], with women with young children being particularly disadvantaged [108].

According to the COR theory mentioned above, women’s increased investment of resources in family and home care is a barrier to career success. In fact, the resources spent on one role put a strain on the other roles, creating a chain reaction. Even though working from home is often cited (at least in Italy) as a work-life balance measure, even before the pandemic the difficulty of working and engaging in family duties at the same time was highlighted, while a benefit of reducing travel time for working from home was noted [109]. In addition, as we have outlined, this aspect was exacerbated during the pandemic, particularly for women.

Moreover, some pre-pandemic studies have highlighted how the dynamics of the work-home interface differ to some extent between women and men [95,110], while differences in recovery experiences warrant further investigation. These differences have been partially confirmed in studies of the first lockdown [58,111], which report lower levels of recovery in women than in men and, conversely, higher levels of exhaustion and concern about the Covid 19 pandemic in the female subsample.

In general, these studies emphasize the importance of multigroup analyses in relation to gender to assess not only differences in individual constructs but also differences in work dynamics [111], also considering the role of technology use [59]. Indeed, the issue of gender difference in remote work is also relevant in relation to technology, because although some studies have shown that technostress in young workers does not vary between women and men [112], other studies have found significant differences [59,113], with women showing higher levels of uncertainty about technology.

In brief, while this is an extreme summary of the main reasons why the issue of gender differences is crucial, it already largely reflects its importance in relation to the proposed model.

## 7. Conclusions

This paper aims to fill a gap in research on middle managerial leadership by discussing some of the potential risks and difficulties (e.g., workaholism and technostress) while considering the bright and dark sides of leadership that may be associated with the work-life interface. Based on the discussion of the existing literature, which already provides a broad starting point for the development of good organizational practices, the paper proposed a theoretical extension that paves the way for future empirical findings. The state of the academic literature to date reveals a fragmented scenario: the relationships between the variables we have studied appear to have been only partially explored. Therefore, a significant research effort is needed to rethink the system of relationships between these variables, both in emergency and in the new normal, in order to help organizations plannig interventions to improve the quality of personal and social life.

The gap to be filled was both theoretical and methodological. From a theoretical perspective, the analysis of the specific characteristics and responsibilities associated with this role led to the call for a renewal of the research framework on the work-life interface, taking into account more dynamic approaches. In line with these findings, Allen and colleagues [114] suggested that more detailed longitudinal studies could be conducted, carefully examining how the work-life interface might change over time and how time perspective itself might be a relevant variable. More generally, it might be useful to expand the literature to include qualitative studies useful for better defining constructs and multilevel studies valuable for increasing the validity of perceptual measures and for testing interaction effects that might also be sensitive to gender differences. Second, a new model of leadership, particularly middle management leadership, needs to be tested to successfully design the virtual work environment into which the “in presence” environment is integrated.

Accordingly, the paper focuses on several research questions. Specifically: How must middle managers interact with their supervisors and subordinates to translate top management strategies into concrete goals for their employees? How can middle managers protect themselves from the new psychosocial risks associated with the negative culture of heavy work investment and technostress? And what if they experienced workaholism and technostress? How can they protect themselves from the harmful effects of a counterproductive and unsustainable work-life interface? And how can they play a positive leadership role in their relationship with their employees to promote work-life balance? Are there differences between men and women in middle management leadership roles in terms of their relationship with the new psychosocial risks and work-life interface?

Answering these questions is therefore a priority for future research in order to: (1) provide useful insights for the development of organizational policies and practices designed to manage the transition to this ‘new normal’ characterized by the ‘always-on’ organizational culture; (2) support a renegotiation of the balance between work and the rest of life; (3) stimulate the development HR of policies and practices aimed at sustainable change management that respects organizational efficiency; (4) raise awareness among stakeholders (organizations and individuals) of the new psychosocial work-related risks, such as workaholism and technostress, in order to prevent such signs and promote well-being at work and subjective well-being.

An initial contribution of this paper was the suggestion to explore both the ‘dark’ (negative) and ‘light’ (positive) sides of leadership—also taking into account the recent inclusion of the ‘positive leadership’ in the psychology section of the Oxford Research Encyclopedias [115]. It would be interesting to review whether the impact of these facets might have a direct influence on the dual interface between work and life (conflict or enrichment), and whether or not these relationships are mediated by the presence of the new psychosocial work risks (workaholism and technostress).

As for the practical implications of the topic of the present study, it is clear that we are facing the “most important technological paradigm shift” [116] (p. 5), so future theoretical investigations should focus on the role of “human control of technology” in order to maintain social equilibrium and create developmental opportunities for all individuals, in line with the goals set out in Art. 3 of Italian Constitution and those published by ONU of Sustainable Development.

In this paper, the main limitations can be attributed, on the one hand, to the literature considered and, on the other hand, to the lines of research proposed. As far as the literature considered is concerned, the main topic and the variables considered in this paper are now the focus of numerous studies that have either just been completed, are in the process of being published or are in progress. We are sure that theoretical knowledge is constantly expanding on an empirical basis, and it is difficult, if not impossible, to give an exhaustive picture of research findings useful for presenting future developments. Moreover, we should be aware that the proposed conceptual model could be better understood against the background of the specific context in which it could be applied. For example, organizational culture should be one of the crucial variables to consider when monitoring the impact of leadership on the work-life interface. We agree that structural and cultural variables, as well as the work sector, should be considered to better fit the proposed conceptual model into a situated perspective.

Regarding the development of research on these issues, cross-sectional studies, although still widely used, are not appropriate for understanding these phenomena. Longitudinal or diaristic studies are needed, which also allow us to understand more complex dynamics. This type of study raises issues of anonymity and privacy, a delicate aspect both from a purely legal point of view and from the point of view of the availability of organizational contexts and individuals for such surveys. Moreover, an in-depth study of the role of middle managers may require the use of data that are not only self-reported, but a combination of self-reported and third-party assessments. This brings up all the “classic” critical issues related to evaluation processes, related to the anonymity and privacy issues mentioned above, but more generally triggering reactions and biases in the research process that require great effort in building the research alliance and a careful process of communicating the goals and data collection process to overcome these biases.

Even with these limitations in mind, starting from the principle of anthropocentrism and a “sustainable” vision of technology that supports economic, cultural and social development (European Committee’s White Paper at Artificial Intelligence and the European Coordinated Plan at Artificial Intelligence), future research would need to focus on the strategic role played by middle management in guiding these changes, and seek to highlight the opportunities that have arisen from the “forced” use of technology that followed the pandemic emergency. The use of remote working could be a tool for individuals, particularly in relation to the need to balance work and life, and it could be a resource for organizations to meet the challenges of very turbulent markets, but only if the use of technology is deliberately and appropriately designed and used, as recently highlighted in the recommendations of the European Agency for Occupational Safety and Health, with particular reference to resumption of work after phase 1 pandemic outbreak [117].
